# Digital interventions to address mental health needs in colleges: Perspectives of student stakeholders

**DOI:** 10.1016/j.invent.2022.100528

**Published:** 2022-03-23

**Authors:** Naira Topooco, Lauren A. Fowler, Ellen E. Fitzsimmons-Craft, Bianca DePietro, Melissa M. Vázquez, Marie-Laure Firebaugh, Peter Ceglarek, Grace Monterubio, Michelle G. Newman, Daniel Eisenberg, Denise E. Wilfley, C. Barr Taylor

**Affiliations:** a Department of Behavioural Sciences and Learning, Linköping University, Sweden; b Center for m^2^Health, Palo Alto University, Palo Alto, CA, USA; c Department of Psychiatry, Washington University School of Medicine, St. Louis, MO, USA; d Department of Health Management and Policy, University of Michigan School of Public Health, Ann Arbor, MI, USA; e Department of Psychology, Pennsylvania State University, University Park, PA, USA; f Department of Health Policy and Management, Fielding School of Public Health, University of California at Los Angeles, Los Angeles, CA, USA; g Department of Psychiatry and Behavioral Sciences, Stanford University School of Medicine, Stanford, CA, USA

**Keywords:** College mental health, Patient perceptions, Stakeholders, Service delivery, Self-help, Stepped care

## Abstract

**Objective:**

The need for clinical services in U.S. colleges exceeds the supply. Digital Mental health Interventions (DMHIs) are a potential solution, but successful implementation depends on stakeholder acceptance. This study investigated the relevance of DMHIs from students' perspectives.

**Methods:**

In 2020–2021, an online cross-sectional survey using mixed methods was conducted with 479 students at 23 colleges and universities. Respondents reported views and use of standard mental health services and DMHIs and rated the priority of various DMHIs to be offered through campus services. Qualitative data included open-ended responses.

**Findings:**

Among respondents, 91% reported having experienced mental health problems, of which 91% reported barriers to receiving mental health services. Students highlighted therapy and counseling as desired and saw flexible access to services as important. With respect to DMHIs, respondents had the most experience with physical health apps (46%), mental health questionnaires (41%), and mental well-being apps (39%). Most were unaware of or had not used apps or self-help programs for mental health problems. Students were most likely to report the following DMHIs as high priorities: a crisis text line (76%), telehealth (66%), websites for connecting to services (62%), and text/messaging with counselors (62%). They considered a self-help program with coach support to be convenient but some also perceived such services to be possibly less effective than in-person therapy.

**Conclusions:**

Students welcome DMHIs on campus and indicate preference for mental health services that include human support. The findings, with particular focus on characteristics of the DMHIs prioritized, and students' awareness and perceptions of scalable DMHIs emphasized by policymakers, should inform schools looking to implement DMHIs.

## Highlights

• This study explored students' perceptions of traditional mental health services and digital mental health interventions (DMHIs)
• Students were enthusiastic about in-person therapy and counseling but noted barriers to access
• Students had typically used DMHIs of a more general nature such as symptom questionnaires. Use of DMHIs for mental health management was low.
• Students welcomed the integration of DMHIs on campuses, particularly those that included human support.
• A self-help program with brief support was seen as convenient and flexible. Some perceived it as less effective than in-person therapy.

## 1 Introduction

The prevalence of mental health problems among American college and university students has nearly doubled in the past decade (Duffy et al., 2019), and the influx of students to counseling centers has strained services and led to long waitlists (The Association for University and College Counseling Center Directors Annual Survey, 2018). The need to identify viable strategies to improve service capacity has been further amplified during the COVID-19 pandemic (Wind et al., 2020; Lederer et al., 2021). A study investigating the prevalence of psychological conditions among U.S. college students a few months before and after the onset of the COVID-19 outbreak reported pre-pandemic prevalence of depression, anxiety (GAD), and suicidal ideation to 39%, 29%, and 21% respectively. A few months later, in the pandemic period, the prevalence of depression was significantly higher, 46% (Kim et al., 2021).

Digital Mental Health Interventions (DMHIs), ranging from self-guided tools for monitoring, assessment, and behavioural skill practice, to complex therapy programs with coach or therapist support, represent an opportunity to scale up mental health resources (Schueller and Torous, 2020; Mohr et al., 2021). Among DMHIs, internet-delivered CBT programs (ICBT), particularly those with coach guidance, have proven effective in many randomized controlled trials (Andrews et al., 2018; Andersson et al., 2014; Carlbring et al., 2018) and have been successfully integrated into health care systems across Europe, Canada, and Australia (Titov et al., 2018). A recent expert consensus statement argued that guided DMHIs with fidelity to core principles of guided ICBT programs should be broadly adopted in the U.S. health care system, given the robust evidence-base for their effectiveness in treating common mental health disorders (Mohr et al., 2021).

While DMHIs hold great potential to scale up mental health resources, implementation of any new health care services is complex and time consuming, and there is also risk of failure (Morris et al., 2011; Mohr et al., 2017a; Mohr et al., 2017b; Mohr et al., 2018). Importantly, acceptance of stakeholders – those who would use, receive, deliver, or otherwise be affected by DMHIs – has been identified as a key determinant for successful integration of DMHIs in real world settings (Mohr et al., 2017a; Vis et al., 2018; Davies et al., 2020). Challenges to client and user engagement with various DMHIs have been observed in studies and implementation efforts (Gilbody et al., 2015; Baumel et al., 2019; Gilbody et al., 2017; Kern et al., 2018; Christensen et al., 2004; Fleming et al., 2018a). To achieve sustainable and successful implementation of DMHIs, further understanding of how and which of such interventions can be aligned with stakeholder needs is warranted (Mohr et al., 2021; Schueller, 2021).

In colleges and universities, students with mental health needs represent key stakeholders. This study aimed to gain an understanding of students' perceptions of DMHIs, and preferences and needs in the campus contexts where DMHIs could potentially be introduced. To capture this, we collected qualitative and quantitative data regarding three inter-related research topics: students' 1) preference and desire for mental health services in a campus setting; 2) adoption, satisfaction, and perceived priority of various DMHIs to be made available through campus mental health services, and 3) viewpoints towards a self-help program with brief coaching support, a type of DMHI with evidence of efficacy and recently pointed out as an implementation priority in the U.S. (Mohr et al., 2021).

## 2 Methods

### 2.1 Procedure

A cross-sectional online survey was conducted with students at 23 colleges and universities across the country. The study was a sub-study conducted in conjunction to a randomized controlled trial (NCT04162847, ClinicalTrials.gov) whose primary aims were to investigate the effectiveness of using online mental health screening and intervention programs with coach support in college populations (Fitzsimmons-Craft et al., 2020). We recruited students from the colleges and universities that participated in the main trial and also agreed to participate in the present study. Study samples did not overlap; at each school, a random sample of 200 students who did not participate in the main intervention trial received email invitations to participate in the present study. Participating schools were diverse across geographic region (5 west, 7 mid-west, 5 northeast, 6 south), institutional type (7 private, 16 public), and school size (2000 to more than 90,000 students). Each school launch followed the timeline of the main trial. At three schools the study was launched twice (i.e., N = 400 invited) in line with the main trial also recruiting on two separate occasions. The only eligibility criterion was being 18 years or older. Participants were presented with an informed consent page prior to starting the survey and were offered $5 for study participation. Procedures were approved by the institutional review board at Palo Alto University, California, U.S.

### 2.2 Questionnaire

Based on a literature review (Gulliver et al., 2010; Dunbar et al., 2018; Topooco et al., 2017; Schuster et al., 2020; Lattie et al., 2019; Renn et al., 2019; Breedvelt et al., 2019; Apolinario-Hagen et al., 2018; Lattie et al., 2020), a questionnaire was designed with aim to suit the target population and study objectives. It included multiple choice and open-ended questions about standard services and 13 different DMHI categories selected, based on being available in the U.S. at the time of the study. The outcomes of interest covered three thematic areas: students' 1) preference and desire for mental health services in a campus setting, including factors that facilitate help-seeking; 2) adoption, satisfaction, and perceived priority of various DMHI categories to be made available through campus mental health services, and 3) further viewpoints towards a self-help program with brief coaching support. Concerning DMHI categories, we included physical health apps as a DMHI category, as for example physical health programs are considered a treatment option for depression (Cooney et al., 2014; Hallgren et al., 2015). Consistent with clients' and clinicians' understanding of DMHIs, we included text and other synchronous messaging as a DMHI (Lattie et al., 2020). We also examined potential technology-related determinants of using DMHIs (e.g., data plans; digital overload (Misra and Stokols, 2011; Smith et al., 2021)). Qualitative data included open-ended comments about standard mental health services and a self-help program with brief coach guidance. Table 1 presents themes and examples of questions. Online supplement 1 presents all questions analyzed.

**Table 1:** Overview of thematic areas and examples of survey items.

Theme	Item	Item design
Standard Mental Health Services	“If you experienced emotional or mental health problems and needed help, how would you wish to receive help and which services would you want available? What kind of service, offered where, and by whom? What would matter the most to you in making it feasible for you to seek help from a professional?”	Open ended
Digital Mental Health Interventions (DMHIs)	“Which of the following e-mental health services are you aware of, or have already tried at the counseling center/health center on your campus, or elsewhere?”	“Not aware of”; “Aware of but never used”; “Previously used”; “Currently using”
	“In your opinion, should e-mental health services be available among other mental health service options to students on your campus?”	“No, not relevant to offer to us students”; “Yes, should be available to us students with *low* priority”; “Yes, should be available to us students with *high* priority”; “I don't know”
DMHI category – self-help program with coaching	“In your opinion, what would be the primary [benefit/advantage] / [shortcoming/challenge] (if any) for students to receive self-help therapy in the form of modules, along with brief online guidance from a professional (~15 min per week), inside an app?”	Open ended

### 2.3 Data analysis

Descriptive statistics were computed using means, percentages, and frequencies. Respondents were allowed to skip any survey questions. Analyses were computed based on the total number of participants with complete data on a given variable. Within the sample, we explored variations by gender identity, sexual orientation, and race. Variables were combined into the following: *gender diverse*, *non-heterosexual, and non-white* (identifying as other than cisgender/heterosexual/white, and/or indicating multiple response options). *t-*Tests, alpha level 0.05, were used to compare difference between groups. Qualitative data: a subset of data (N = 146 respondents who commented in response to one or more open-ended questions) were analyzed using Consensual Qualitative Research-Modified methodology (Spangler et al., 2012), a method that can be used in studies collecting large amounts of relatively brief and less complex qualitative data. Authors N.T., L.F., B.D., and M.V. analyzed and coded the data through an iterative sorting process. First, coders read all responses, then independently categorized responses. Next, they met to review and to arrive at one list of domains (major themes) and categories (subthemes). In cases of discrepancies, consensus was reached by discussion. N.T., B.D., and M.V. independently coded responses to confirm favorable agreement, with L.F. serving as auditor. Krippendorff's alpha coefficient (Hayes and Krippendorff, 2007) was used to test inter-rater reliability.

## 3 Results

### 3.1 Sample

Between April 15, 2020, and July 29, 2021, a total of 570 (11.0%) individuals consented to the survey. Of those, 84.0% (N = 479) completed at least one item related to the study objectives and were included in the analyses (Table 2). Respondents (M = 20.8 years, SD = 4.25) predominately identified as White (55.0%), Non-Hispanic (80.0%) and female (63.8%). Nine of ten (90.6%) endorsed having experienced emotional and mental health problems (average number of problems experienced M = 3.49 [SD = 1.29, N = 434]). Among those, 52.8% reported that they had received mental health services (counseling, therapy, or medication) for their problems. Respondents who had received mental health services reported overall neutral to moderate satisfaction with these; M = 3.41 (SD = 1.09, N = 228). Among respondents who endorsed mental health problems, 90.8% reported that they had encountered barrier(s) in accessing mental health services. The top barrier was “Prefer to deal with issues on my own” (45.9%, N = 199). Identifying as non-heterosexual was associated with endorsing more barriers (M = 3.03, SD = 1.76; N = 116) than identifying as heterosexual (M = 2.47, SD = 1.67, N = 291), t = 3.02, *p* = 0.003.

**Table 2:** Baseline characteristics of participants.

Characteristics (% of total sample)	N	%
Age (M)	479	20.8
Gender identity		
Female	272	63.8
Male	136	31.9
Gender diverse	18	4.2
Year in school		
Undergraduate	333	94.1
Graduate/other	21	5.9
Sexual orientation		
Heterosexual	332	73.6
Non-heterosexual	119	26.4
Race		
White	214	55.0
Non-White	142	36.5
Do not wish to disclose or do not know	33	8.5
Ethnicity		
Non-Hispanic	340	80.0
Hispanic	85	20.0
Mental health problems ever experienced		
Any^a^	434	90.6
Stress	380	79.3
Anxiety	341	71.2
Depression	283	59.1
Sleeping problems	242	50.5
Eating/weight concerns	233	48.6
Other	35	7.3
Received mental health services^b^		
Yes^a^	229	52.8
No	205	47.2
Experienced barrier(s) to care^b^		
Any	394	90.8
Prefer to deal with issues on my own	199	45.9
Not enough time	153	35.3
Not sure where to go	146	33.6
Financial reasons (too expensive, not covered by insurance)	137	31.6
Don't want anyone to know	105	24.2
No need for services	88	20.3
Difficulty finding an available appointment	81	18.7
Need to obtain parental consent^c^	74	18.1
Prefer to deal with issues with support from family/friends	69	15.9
Takes too long to get help	51	11.8
Other	31	7.1

Note. Percentages were computed based on the total number of participants with complete data on a given variable.

a Variables collapsed: “Yes prior to college”, “Yes, since starting college”, “Yes both prior/since college”.

b Calculated for those who endorsed ever experiencing mental health problem(s) (N = 434).

c The item was presented separately in the survey.

### 3.2 Desires and facilitating factors for mental health services

Respondents were asked to describe how they would want to receive mental health services (what kind of service, offered where, by whom), and what would matter the most in making it feasible to seek help from a professional. Participants' open-ended responses (N = 111) were analyzed and coded into lists of domains. Domains/themes were not mutually exclusive; one participant response could be coded into several domains. Online Supplement 1 presents all domains and categories.

The most common type of service participants wanted was “therapy/counseling” (42.3% of responses, N = 47), which included therapy, counseling, mental health professionals and psychologists. The theme with the second most responses was “near/on-campus” (18.9%, N = 21), e.g., for services to be located close by campus, or in walking distance. Regarding what would matter in making it feasible to seek help, the most identified facilitator was “flexibility” (34.2%, N = 38), which included access to care outside of normal work hours, reduced wait times, possibility for “walk-ins”, and online alternatives. The next most common perceived facilitating factors identified were “social connection” (18.0%, N = 20; e.g., support from friends/family, sharing gender, race or age with providers, or being able to trust providers), and “campus climate” (18.0%, N = 20; e.g., mental health being a priority on campus, advice from school on where and how to receive help, databases of mental health professionals and what insurance they take).

### 3.3 Awareness and adoption of DMHIs

**Fig 1:**
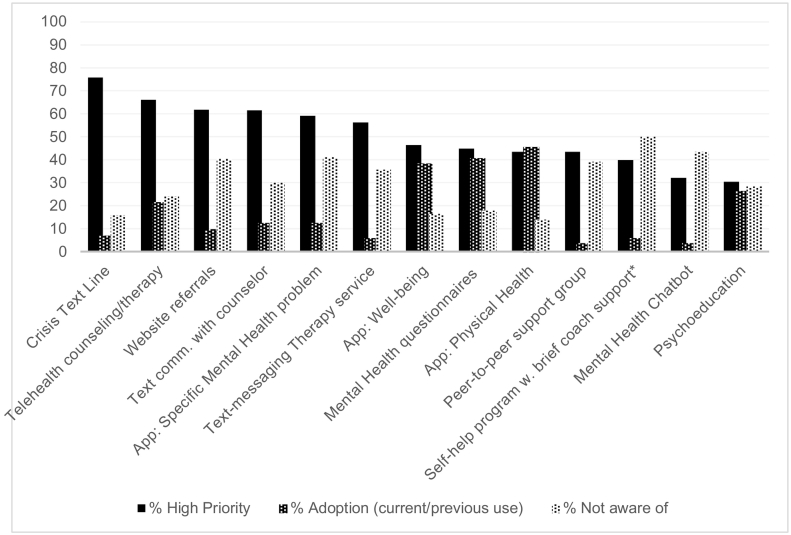
Digital Interventions to Address Mental Health Needs: Awareness, Adoption, and Perceived Priority among U.S. college students^a^. Note. Sample sizes: Awareness/Adoption, N = 438–457; Priority, N = 374–398. ^a^Responses were coded dichotomously for descriptive purposes: “High priority”, versus other responses; “DMHI use” versus other responses; “Not aware of DMHI” versus other responses. Previous and current DMHI use were collapsed into “Adoption”. *The item was presented separately in the survey.

Fig. 1 presents results for the 13 DMHI categories. Awareness was defined as endorsing DMHI use or awareness, as opposed to ‘not aware’. On average, respondents were aware of M = 8.95 (SD = 3.77, N = 459) of the 13 DMHIs. They were most familiar with crisis text lines (84.0%, N = 382 of 455) and least familiar with self-help therapy programs with brief coach-support (50.0%, N = 219 of 438). Among respondents, 73.6% (N = 338 of 459) reported current/previous use of at least one DMHI category. The top three DMHIs adopted were physical health apps (45.6%, N = 208 of 456), online mental health screens/questionnaires (40.8%, N = 186 of 456), and mental well-being apps (38.5%, N = 175 of 455). Identifying as non-heterosexual was associated with using more DMHIs (M = 3.71, SD = 2.34; N = 119) than identifying as heterosexual (M = 1.84, SD = 1.96, N = 316), t = 8.33, *p* < 0.001. Identifying as male was associated with using less DMHIs (M = 1.45, SD = 1.84, N = 132) than identifying as female (M = 2.66, SD = 2.31, N = 258), or gender diverse (M = 3.78, SD = 1.70, N = 18), respectively, t = 5.22; t = 5.07, *p*s < 0.001. Satisfaction ratings were obtained from respondents who endorsed DMHI use. By type of DMHI, highest satisfaction was reported for telehealth (M = 3.78, SD = 0.94, N = 65), and lowest satisfaction for chatbots (M = 2.33, SD = 1.21, N = 6) (Table 3).

**Table 3:** Digital interventions to address mental health needs: satisfaction among U.S. college students.

Category	N^a^	Mean^b^	(SD)
Telehealth counseling/therapy	65	3.78	0.94
Text communication with counselor (messaging, chat)	32	3.66	1.00
App for physical health	124	3.66	0.86
App for general mental well-being	108	3.61	0.94
Digital peer-to-peer support group	7	3.57	0.78
App for specific mental health problem	34	3.50	0.96
Psycho-education material (e.g., Mayo Clinic, YouTube)	76	3.37	0.96
Questionnaires for mental health	109	3.34	0.75
Text-messaging therapy service (e.g., Talkspace)	15	3.33	0.90
Website connecting with services (e.g., 211.org)	20	3.25	1.02
Crisis text line service	17	3.24	1.20
Self-help program w. brief coach support	22	3.13	0.99
Mental health chatbot	6	2.33	1.21

Note. 112 respondents provided an overall DMHI satisfaction rating and were removed from analyses.

Five-point Likert scale, from “1 = Very unsatisfied”, to “5 = Very satisfied”.

a Number answering the question by type of DMHI.

b Mean satisfaction scores for DMHIs were calculated based on ratings for previous/current DMHI use.

### 3.4 Priorities of DMHIs

The DMHIs most frequently identified as high priorities to be offered at campuses were crisis text line (75.7%, N = 296 of 391), telehealth counseling/therapy (66.0%, N = 258 of 391), websites for connecting to services (61.8%, N = 244 of 395), and text/messaging with counselor (61.5%, N = 241 of 392). On average, respondents identified M = 6.46 (SD = 4.096, N = 400) DMHI categories as high priority to offer on their campus. Identifying as male was associated with selecting less DMHIs as high priority (M = 4.76, SD = 3.99, N = 117), compared to identifying as female (M = 7.34, SD = 3.88, N = 236, t = 5.81, *p* < 0.001), or gender diverse (M = 7.75, SD = 3.25, N = 16, t = 2.86, *p* = 0.05).

### 3.5 Perceptions of a DMHI with implementation priority

Related to the primary study (Fitzsimmons-Craft et al., 2020), the survey explored respondents' expectations and perceptions of a self-help therapy program with brief coach-support.

*Interest and compliance*. Among respondents, 22.1% (N = 96 of 435) stated that a self-help therapy program with brief coach-support was something they would personally want to try for their mental health; 52.2% stated ‘Maybe’, or ‘I don't know’, and 25.7% stated ‘No’. When asked if they would try this type of DMHI for their mental health if recommended/available from the campus counseling or health center, 51.2% (N = 191 of N = 373) ‘Agreed’, or ‘Strongly Agreed’, as opposed to being neutral/not agreeing.

*Perceived benefits and shortcomings.* Participants provided open-ended responses to two questions related to the perceived benefits and shortcomings of this DMHI. Responses were coded for each question separately and could be coded for mentioning more than one theme. Online Supplement 1 presents all domains and categories.

Benefits (N = 94): The most common advantage identified was “convenience” (43.6% of responses, N = 41, e.g., ‘Being able to do it at any time they need. Including at night.’), which included increased reach, affordability, and access anytime and anywhere. The next most common advantage was “flexible” (26.6%, N = 25, e.g., self-paced, fitting within one's schedule, or “a good first step”), and “symptom improvement” (22.3%, N = 21, e.g., “reduced stress, overall morale boost”).

Shortcomings (N = 89): The most common perceived shortcoming was “relative effectiveness” (49.4%, N = 44), which included expectations of the DMHI being inferior to in-person care and/or impersonal. The second and third most common shortcomings identified was “accountability” (20.2%, N = 18, e.g., lack of accountability to a person, and/or having no set appointment times), and “time/effort” (N = 16, 18.0%, e.g., perceived lack of time to use the DMHI; the DMHI adding to workload or high effort).

*Potential technology-related determinants of use.* Among respondents, 25% (N = 93 of 372) ‘Agreed’ or ‘Strongly Agreed’ that inadequate smartphone access or storage capacity/data plan would make it difficult for them to use the DMHI, and 46.2% (N = 171 of 370) ‘Somewhat Agreed’ or ‘Completely Agreed’ that being overloaded by digital information, e.g., keeping up with/being distracted by emails, social media, and notifications, would make it hard for them to use the DMHI.

## 4 Discussion

This study informs the conditions for the implementation of DMHIs in colleges and universities by providing insight into student stakeholders' views on standard mental health services and a range of DMHIs, by type of service.

Nine out of ten students reported that they had experienced mental health problems; of those, about half had received traditional mental health services. In terms of DMHIs, students mainly reported experience with physical health apps, mental health questionnaires, and general mental wellbeing apps. While these categories are relevant in terms of identifying and improving mental health they are of a more general nature. It was less common to have experience with DMHIs that focus more explicitly on improvement or management of mental health problems. For example, most students said they were unaware of apps for depression, anxiety, or body image, or they had not used them, leaving a minority who reported that they had used them (12.4%). The finding is noteworthy given the high prevalence of mental disorders among college students (Kim et al., 2021), the ubiquity of mental health apps available to treat depression and anxiety, and students' extensive use of smartphones, including apps for numerous purposes. Similarly, experience with a self-help program with brief coach support, a type of DMHI with considerable evidence-base, was modest. Recently, U.S. college students have described using games, entertainment apps, and social media apps to manage their mental health, as opposed to, for example, evidence-based apps targeting specific problems (Smith et al., 2021). Students' views and uptake of DMHIs may be different and extend beyond evidenced-based DMHIs prioritized by policy makers.

Even though few students reported using DMHIs for mental health problems, they identified several of them as being of high priority to offer on their campus. Among DMHIs, students showed particular interest in DMHIs characterized by enabling or including support from a trained supporter or specialized mental health provider –– e.g., crisis text line, telehealth, and text communication with a counselor. In the scientific literature DMHIs are divided into two overarching categories; guided DMHIs, characterized by enabling or including support, and self-guided DMHIs that users use entirely on their own (Schueller and Torous, 2020). Self-guided DMHIs are considered to have the highest potential in terms of cost-effectiveness and scalability (Muñoz et al., 2015) whereas guided DMHIs have been observed to fare better in terms of clinical efficacy and engagement (Grist et al., 2019; Karyotaki et al., 2021; Baumeister et al., 2014), and stakeholder acceptance (Davies et al., 2020; Topooco et al., 2017). The finding of students' interest in DMHIs with human support is in line with previously reported findings, which have included preference among stakeholders for in-person therapy and blended treatments (combination of sessions and self-help) over guided ICBT programs (Davies et al., 2020; Topooco et al., 2017; Peynenburg et al., 2020; Lokkerbol et al., 2019; Gericke et al., 2021). In our study we did not categorize and directly compare DMHIs based on guidance/self-help so as not to introduce bias in students' reflections on them. Nonetheless, students' priority ratings were aligned with such a classification, highlighting that for students, those preferences may apply across a range of DMHIs.

Related to our goal to assess students' perceptions and attitudes towards a specific type of DMHI with well-established evidence of efficacy— self-help programs with brief support—students again noted a preference for human support. Although they noted that the DMHI may be convenient and accessible they compared it with in-person therapy and counseling services and expected it to be impersonal or possibly not as supportive (Online suppl.). Gericke and colleagues recently found similar views among South African students based on first-hand experiences with this type of DMHI (Gericke et al., 2021).

Student preferences for some human support are a finding with implication. Colleges and universities now provide unguided, pure self-help programs, perhaps, as an inexpensive alternative to more labor-intensive programs, but the benefits of self-help alone remain uncertain, and engagement is low (Fleming et al., 2018b). Thus, colleges and universities may need to consider other models: for instance, stepped-care models (Wilfley et al., 2013; Cornish et al., 2017; Bower and Gilbody, 2005).

Last, concerning our goal to map preferences and needs for existing mental health services, students in our study highlighted in-person therapy/counseling and indicated limited access to existing services. In response to the question of what would matter the most in enabling them to seek professional help, they suggested more liberal operating hours, more meeting options (online, on/near campus), and information from the school about what types of services are available (Suppl. 1). This finding suggests that students would be more likely to seek therapy if there was improved capacity and access to mental health services, a finding somewhat different from previous studies with college students that suggest they do not seek mental health services mainly because of personal or attitudinal barriers (Ebert et al., 2019; Eisenberg et al., 2011). Relatedly, it is possible that barriers to alternatives of traditional treatment experienced by students may inflate interest in DMHIs. It cannot be ruled out that students' high interest for human-centered alternatives, reflect that they may view DMHIs as pathways to professionals rather than as stand-alone options for self-care.

This study recruited from diverse schools in terms of geographic region and institutional type. On the participant level, we sampled broadly (not limiting to e.g., first-year students) at random. Results should be interpreted with care as a limited number of students responded to the survey. Whereas the high rates of mental health concerns in the included sample may add credibility to the findings about mental health service needs, views may not reflect those of a general population and the assessment relied on self-report and not validated measures. While investigating a range of DMHIs, the survey may not have captured all DMHIs perceived as priorities among students. Furthermore, the DMHIs investigated were described briefly, and students might have had somewhat different understandings of these services. For the physical health app category, we did not specify as a requirement that the use be intended to improve mental health specifically. Recruitment during COVID-19 pandemic may bias findings, for example, students' perceptions may have changed over time in relation to the pandemic.

### 4.1 Conclusion

According to a national sample of U.S. students, DMHIs have a large potential role as part of campus mental health services, with priority for alternatives that include human support. Students seem to not differentiate when and how scalable self-help and guided DMHIs programs may be suitable compared to face-to-face therapy. Given the many advantages of such services in terms of access, continued work to explore students' expectations regarding DMHIs, standard mental health services, and the relationship between the two, is imperative to understand how students' desires can be addressed. Effects of communication that emphasize relative advantages identified by students themselves, in this study and elsewhere (Peynenburg et al., 2020) –– access, convenience, and flexibility, should be further investigated. As part of this it might be useful to include students in considering services, with open discussions about stepped care models where DMHIs are offered as no-delay options. Schools may also consider introducing DMHIs as adjuncts to or blended with in-person services to facilitate acceptance and knowledge.

## Funding

This study was supported by the 10.13039/100000025National Institute of Mental Health (R01 MH115128). Dr. Topooco is supported by the 10.13039/501100004359Swedish Research Council (2018-06585); Dr. Fowler is supported by the 10.13039/100000050National Heart, Lung, and Blood Institute (T32 HL130357); Dr. Fitzsimmons-Craft is supported by the 10.13039/100000002National Institute of Mental Health (K08 MH120341). The funding sources of this study had no role in study design, data collection, data analysis, or data interpretation.

## Declaration of competing interest

The authors declare that they have no known competing financial interests or personal relationships that could have appeared to influence the work reported in this paper.

## Appendix A Supplementary data

Supplementary material Image 1
