# Supplementary material for: Digital interventions to address mental health needs in colleges: Perspectives of student stakeholders
Source: Internet Interv. 2022 Mar 23;28:100528. doi: 10.1016/j.invent.2022.100528 (PMC8976123; doi:10.1016/j.invent.2022.100528)
Supplement: Supplementary file 1 — Supplementary material [file mmc1.docx]

**Qualitative results**

**Box 1.** Standard Mental Health Services: Preferences and Identified Facilitating factors among U.S. college students (N=111).

| Domains and Categories | Category definition and example(s) | %^a^ | F^b^ |
| --- | --- | --- | --- |
| Services by whom/ where |  |  |  |
| Therapy/counseling | Therapy, counseling; mental health professional; psychologist   - ‘I wish actual therapists who care about your mental health were available. When I went, my issues were brushed off as something that everyone has’ - ‘Therapy and being able for someone to be available to listen to me would really help. I believe the help offered on campus is well prepared to help me with those tasks’ - ‘I would want access to routine one-on-one meetings with a therapist.’ | 19.92 | 47 |
| Near/on-campus | Close proximity; walking distance; on campus; offered by school   - ‘Professional psychologists for therapy located at close places to campus. It needs to be easy to make an appointment and cheap’ | 8.90 | 21 |
| Online services | Online services; phone, or video call; text chat system for support between appointments   - ‘A like, quick chat with a professional, in text or online would be awesome. Like if I felt I had a one time issue that was somewhat urgent, and couldn't wait to get an appointment with CAPS’ - ‘If I could email someone and talk to them a bit before meeting them in person for counselling that would be great’ - ‘Online counseling sessions may be helpful, since they cannot be done in person [because of the Covid-19 pandemic]. I found talking/therapy to be the most helpful’ - ‘Virtual meetings, even when we’re back on campus.’ | 5.08 | 12 |
| Self-management or help from friends/family | Social connection/someone to talk to; friends/family | 4.24 | 10 |
| Mental health workshops or non-traditional | Non-traditional treatment (e.g., therapy dogs); clubs, sports; mental health classes or events; events or lounges to connect with others experiencing similar issues | 3.81 | 9 |
| In-person | Physically meeting; in person; face-to-face private room | 2.97 | 7 |
| Off-campus help | help off campus/providers in the area; non-campus affiliated; | 3.60 | 4 |
| Psychiatric help | Medication (if needed); psychiatric help | 1.27 | 3 |
| Facilitators |  |  |  |
| Flexibility | Scheduling and walk-in flexibility (more hours and non-traditional times to account for mental health episodes that occur outside of ‘normal’ hours), reduced wait times; access – capacity/resources; quick chat; email contact; in short breaks in between classes; virtual counseling/therapy or meetings; onsite/close to campus; easily accessible at all times; easier to get appointments; mental health app   - ‘I would like there to be longer drop in hours and possible a phone number to call to talk to someone after the counseling center closed.’ - ‘services should have way more availability to students. there’s never time of space or you have to wait forever’ - ‘I wish that counseling went longer than 8-4pm on campus; sometimes I have anxiety attacks at 9pm and there is no one for free I can talk to; lest I'm feeling suicidal.’ - ‘[…] What matters most to me is how close these services are to my campus. Without a car, it is difficult to go to therapy or counseling, and it's also difficult to fit in an appointment with a busy school schedule.’ | 16.10 | 38 |
| Social Connection | Connection with therapist/therapist who cares, someone you trust; gender, race; age of counsellor; friend-support to seek help   - ‘I would just want someone to talk to like a therapist, preferably someone of the same race or gender (or both) as me in order to feel more comfortable opening up about what’s on my mind.’ | 8.47 | 20 |
| Campus climate | Mental health everyday priority on campus, clear advice from school on where and how to receive help, databases of mental health professionals for students listing what insurance they take; information and personal guidance on where to turn and what kind of services   - ‘I would want to see a professional psychiatrist, therapist, or psychologist depending on the problem. I think having a database with a list of these professionals in the area with the health insurance they accept would be helpful. It is frustrating having to ask around to find one’ - ‘First, a school that is transparent and vocal to their students about where to go and how to receive help on campus is very necessary.’ - ‘[…] What would make it the most feasible is the creation of a campus climate where mental health is seen as a priority & students have easy access to the knowledge that resources exist […]’ | 8.47 | 20 |
| Competence/specialized | Competence/specialized   - ‘I would like therapy that does not involve simply recommending me to off-campus care but includes having adequate care on campus.’ | 5.51 | 13 |
| Affordable | Cost/covered by insurance, affordable   - ‘I would want an affordable way to receive help with my mental health problems.’ - ‘I would see getting therapy as a burden on myself and my parents if I had to pay for it. I'm a money oriented individual, and if a therapy visit costs $25 without me seeing it as worth $25, I won't do it anymore.’ | 4.66 | 11 |
| Privacy | Confidentiality/privacy; anonymity   - ‘I would wish to receive help anonymously’ | 2.97 | 7 |
| Increased mental health literacy or reduced stigma | Reduced stigma-related barriers, decreased stigma, know problems are ‘enough’ to seek help; assist with psychoeducation   - ‘Counseling would be great. I think anonymity/confidentiality would make it feasible for me to seek help. I’m too worried about others finding out and thinking I’m crazy’ - ‘[…] What stopped me from seeking help early on, was the shame and lack of understanding from my loved ones. I felt alone and that I would waste people's time over feelings that I shouldn't be feeling. I think a great way to help with this problem is to spread more awareness about mental health. At my school we have to attend a one day class about violence, and sexual assault, and I think it would be a good thing to implement a similar class but pertaining to mental health.’ - I feel like the stigma against mental health has caused a great barrier between me and my desire to get help. Additionally, I feel like my problems aren't enough of a burden to warrant seeking help’ | 2.54 | 6 |
| No need for services |  | 3.39 | 8 |
| Total |  | 100 | 236 |

Krippendorff’s alpha ranged from .47 to 1 for the thematic coding of categories with n ≥ 10 responses, with overall thematic message coding rated as moderate (alpha=0.74, 95 CI=0.51 to 0.95). The number of codes per response ranged from 1 to 6 with an average of 2.12 (SD=1.24) codes assigned to each response.

^a^Percentage by which this category, and domain appeared across responses.

^b^Frequency of responses per category and domain.

**Box 2.** Expected benefits of a self-help therapy program with brief coach support among U.S. college students (N=94).

| Domains and Categories | Category definition and example(s) | %^a^ | *f*^b^ |
| --- | --- | --- | --- |
| Modality Benefits |  | 60.87 | 84 |
| Convenience | Access anytime, anywhere; Faster/no wait time; Access in a crisis; Easy access with a device; Effortless/easy way to get help; Reaches more people; Affordable/reduced cost   - ‘They would benefit from this because they wouldn't have to travel/walk/go to ay appointments, they can participate in this wherever they are or if they are on-the-go, which is the case for most college students’ - ‘Being able to do it at any time they need. Including at night’ - ‘It makes it easy for students to get help instead of getting help when it's too late’ | 29.71 | 41 |
| Flexible | Self-paced/flexible schedule; Fit in with daily life/ routine; Regular help and not long/saves time; Resource options; Helpful for those who prefer independence/ aid autonomy; A first step   - ‘It is very self regulated and may be an easy access source in times of high need with low resources available (middle of the night, weekend, etc.)’ - ‘It would be easier for busy college students to fit it into their schedules’ | 18.12 | 25 |
| Integrity | Privacy/discretion; Anonymous; Secure   - ‘Would offer more privacy’ - ‘Make it anonymous’ | 7.25 | 10 |
| Stigma | Less intimidating; Reduced barriers regarding seeking in person treatment, Decrease in structural thresholds to care/equality; Reduced stigma; Discrete:   - ‘It sounds easy and private, so i think for many people who are struggling with mental health who typically feel overwhelmed and want to hide, this is ideal’ | 5.80 | 8 |
| Behavioral, Mental, Social Benefits |  | 31.16 | 43 |
| Symptoms Improvement | Reduced stress/calmer mindset; Help with feeling overwhelmed   - ‘15 minutes adds up and could provide some kind of long term therapy. Or a quick mental health break could prevent a panic attack from spiraling.’ - ‘Being able for students to learn how to relax.’ - ‘Help students that feel overwhelmed’ - ‘Reduced stress, overall morale boost’ | 15.22 | 21 |
| Awareness/ Literacy | Self-awareness/self-understanding; Mental health literacy; Mental health check-in/ keeping track of mental health   - ‘It seems as though it would leave a lot of introspection to the user, which could be helpful to improving their understanding of themselves and their struggles’ - ‘Easy check ins to see if their mental health is increasing or decreasing’ - ‘keep track of mental health’ | 5.80 | 8 |
| Sharing/Connection | Get things off chest; Not being alone/outlet to solve problems; Connect with someone; Personalized guidance   - ‘Let them talk to others about problems’ - ‘You'd get to talk with someone and get things off your chest’ - ‘Having easy access to talking someone’ | 5.80 | 8 |
| Routine/ Self-care | Structure and routine; Prioritization of self-care; Increased frequency of intervention   - ‘good to make time and schedule into their day’ - ‘the routine of it would be nice’ - ‘Set routine for coping healthily’ | 4.35 | 6 |
| Minimal Benefits | Inferior [to traditional modalities]; too brief; not enough guidance; too general/not individualized;   - ‘I think talking to a real person in important’ - ‘self-help therapy is probably good for some people, but it's not good for me. I need to connect with a person and feel like they understand me, not feel like one of may on their roster. That makes me feel like I'm really just pretending I'm not alone when I am’ | 7.97 | 11 |
| Total |  | 100 | 138 |

Krippendorff’s alpha ranged between 0.79-0.91 for the thematic coding of categories with n≥10 responses, with overall thematic message coding for perceived benefits rated as good (alpha=0.79, 95 CI=0.60 to 0.93). Further, while each response could have been coded with 1-9 categories, 93.6% (n=88) of responses contained two or less codes.

^a^Percentage by which this category, and domain appeared across responses.

^b^Frequency of responses per category and domain.

**Box 3.** Expected shortcomings of a self-help therapy program with brief coach support among U.S. college students (n= 89).

| Domains and categories | Category definition and example(s) | %^a^ | f^b^ |
| --- | --- | --- | --- |
| Relative effectiveness | Inferior to in-person/traditional services, non-personalized, less effective, dosage too short   - ‘It just is not able to transcend the non-tangible factor for me. Being in counseling is walking into a building, sitting in a room and sharing space with a human being who is listening, breathing and acknowledging you. In seeking mental health counseling, I want the best, not a substitute or band-aid’ - ‘it loses a bit of humanity; one of the main points of therapy is that you are there; person to person, airing out your grievances. Without that human contact, it kinda feels like your being shoved off, as if you're just another number’ - ‘15 minutes isn't very long, and modules/online courses often feel redundant and impersonal’ | 42.72 | 44 |
| Relative competitiveness |  | 51.46 | 53 |
| Accountability | Lack of scheduled time and personal accountability   - ‘It's difficult to keep up with something like that when there's no consequence to skipping a day, and that can be a problem for people with poor mental health who may have trouble keeping up with daily schedules’ - ‘It is easy to stop doing and forget about doing it when it's an app. Scheduled meeting times make it more of a priority’ | 17.48 | 18 |
| Time/Effort | Perceived lack of time, perceived as work or high effort   - ‘Could easily get lost in the clutter of life since so many people have so much going on’ - ‘I think they would feel it would add to their workload’ | 15.5 | 16 |
| Misc. | Cost, privacy, stigma, logistical barriers   - ‘Difficulty in logistics of it’ - ‘Getting people to open up and be honest about what they are feeling’ - ‘people might judge if they see it, it might not be available during their free time’ | 11.65 | 12 |
| Technology | Barriers specific to technology use, e.g., technology literacy, data plans   - ‘Not tech savvy’ - ‘Those with a lack of technology who can't access the internet very well’ | 6.80 | 7 |
| N/A barriers |  | 5.83 | 6 |
| Total |  | 100 | 103 |

Note. Two nonsensical responses removed.

Krippendorff’s alpha ranged between 0.78-.87 for the thematic coding of categories with n ≥ 10 responses, with overall thematic message coding for perceived shortcomings rated as good (alpha=0.84, 95 CI=0.71 to 0.95). Further, while each response could have been coded with 1-6 categories, 85.4% (n=76) of responses contained only one code.

^a^Percentage by which this category, and domain appeared across responses.

^b^Frequency of responses per category and domain.

| **Supplementary Table 1. Survey items** | |  | |  |
| --- | --- | --- | --- | --- |
| **Theme** | **Item** | | **Item design** | |
| **Standard Mental Health Services** | “Have you ever received counseling, therapy, or medications for mental health concerns that you have experienced?” | | Single choice; “No, never”; “Yes, prior to starting college”; “Yes, since starting college”; “Yes, both of the above (prior to college and since starting college)” | |
|  | “Please provide a rating for your overall experience with the mental health intervention(s) you have received” | | 5-point Likert scale:  1=Very Unsatisfied to 5=Very Satisfied | |
|  | ”Have any of the following factors ever caused you to receive fewer services (e.g., counseling, therapy, or medications) for your mental or emotional health than you would otherwise have received?” | | Select from a list | |
|  | “Before you turned 18, did the need to obtain parental consent ever cause you to receive fewer services (e.g., counseling, therapy, or medications) for your mental or emotional health than you would otherwise have received?” | | Single choice: “Yes”; ”No, parental consent did not cause me to receive fewer services”; No, I didn’t need mental services when I was <18 years”; “I don’t know” | |
|  | ”If you experienced emotional or mental health problems and needed help, how would you wish to receive help and which services would you want available? What kind of service, offered where, and by whom? What would matter the most to you in making it feasible for you to seek help from a professional?” | | Open ended | |
| **Digital Mental Health Interventions (DMHIs)** | ”Which of the following e-mental health services are you aware of, or have already tried at the counseling center/health center on your campus, or elsewhere?”^1^ | | Single choice: “Not aware of; “Aware of but never used”; “Previously used”; “Currently using” | |
|  | “Coached mobile mental health platforms are used to provide self-help therapy programs through self-help modules, along with brief online guidance from a professional (~15 minutes per week). Clients download a mobile app to start the intervention. Are you aware of, or have already tried, any mobile or app-based self-help therapy program at the counseling center/health center on your campus, or elsewhere?” | | Single choice: “Not aware of; “Aware of but never used”; “Previously used”; “Currently using” | |
|  | “You indicated current /previous use of [DMHI category]. Please provide a rating for your overall experience with the service”. | | 5-point Likert scale: 1=Very Unsatisfied to 5=Very Satisfied | |
|  | ”In your opinion, should e-mental health services be available among other mental health service options to students on your campus?”^1^ | | Single choice: “No, not relevant to offer to us students”; “Yes, should be available to us students with *low* priority”; “Yes, should be available to us students with *high* priority”; “I don’t know” | |
| **DMHI category – self-help program with coaching** | ”Reflecting on the description above, if you needed of mental health support, do you think a mobile or app-based self-help therapy intervention is something you would want to try (again)?” | | Single choice: “No”; “Maybe”; “Yes”; “I don’t know” | |
|  | “If you experienced mental health challenges, would you use a mobile or app-based self-help therapy intervention if recommended /available to you from the counseling/health center on your campus?” | | 5-point Likert scale: Strongly Disagree (1), to Strongly Agree (5) | |
|  | ”In your opinion, what would be the primary [benefit/advantage] / [shortcoming/challenge] (if any) for students to receive self-help therapy in the form of modules, along with brief online guidance from a professional (~15 minutes per week), inside an app?” | | Open ended | |
|  | ”If you were recommended to download an app-based (or mobile) self-help therapy intervention, would smartphone access or adequate storage capacity/data plan make it difficult for you to access the service?” | | 5-point Likert scale: Strongly Disagree (1), to Strongly Agree (5) | |
|  | ”To what extent do you agree with this statement: Being ”overloaded” by other competing digital information/ communication would make it hard for me to regularly use an app/internet mental health program that might benefit me?” | | 5-point Likert scale: Completely Disagree (1), to Completely Agree (5) | |

^1^ “Online psycho-education material (e.g. YouTube videos, Mayo Clinic, WebMD)”; “Websites connecting people with mental health services and resources (e.g. 211.org)”; “App for general mental well-being (e.g. meditation, sleep, relaxation)”; “App for specific mental health problem (e.g. depression, anxiety, body image)”; “App for physical health (e.g. fitness/nutrition, medical reminder, smoking)”; “Online questionnaires for mental health screening/assessment/diagnosis”; “Text communication (text messaging, chat, instant messaging with counselor)”; “Counseling/therapy using telehealth (secure videoconferencing software)”; “Counseling/therapy text messaging service (e.g. Talkspace)”; “Crisis text line service”; “Digital peer-to-peer support group”; “Mental health chatbot/conversational agent”.
